# Systematic genomic and translational efficiency studies of uveal melanoma

**DOI:** 10.1371/journal.pone.0178189

**Published:** 2017-06-08

**Authors:** Chelsea Place Johnson, Ivana K. Kim, Bita Esmaeli, Ali Amin-Mansour, Daniel J. Treacy, Scott L. Carter, Eran Hodis, Nikhil Wagle, Sara Seepo, Xiaoxing Yu, Anne Marie Lane, Evangelos S. Gragoudas, Francisca Vazquez, Elizabeth Nickerson, Kristian Cibulskis, Aaron McKenna, Stacey B. Gabriel, Gad Getz, Eliezer M. Van Allen, Peter A. C. ‘t Hoen, Levi A. Garraway, Scott E. Woodman

**Affiliations:** 1 Department of Medical Oncology, Dana-Farber Cancer Institute, Harvard Medical School, Boston, Massachusetts, United States of America; 2 The Broad Institute of Harvard and MIT, Cambridge, Massachusetts, United States of America; 3 Ocular Melanoma Center and Retina Service, Massachusetts Eye and Ear, Department of Ophthalmology, Harvard Medical School, Boston, Massachusetts, United States of America; 4 Orbital Oncology and Ophthalmic Plastic Surgery Program, Department of Plastic Surgery, The University of Texas MD Anderson Cancer Center, Houston, Texas, United States of America; 5 Department of Systems Biology, The University of Texas MD Anderson Cancer Center, Houston, Texas, United States of America; 6 Department of Melanoma Medical Oncology, The University of Texas MD Anderson Cancer Center, Houston, Texas, United States of America; 7 Department of Genome Sciences, University of Washington, Seattle, Washington, United States of America; 8 Department of Human Genetics, Leiden University Medical Center, Leiden, The Netherlands; Rutgers University, UNITED STATES

## Abstract

To further our understanding of the somatic genetic basis of uveal melanoma, we sequenced the protein-coding regions of 52 primary tumors and 3 liver metastases together with paired normal DNA. Known recurrent mutations were identified in *GNAQ*, *GNA11*, *BAP1*, *EIF1AX*, and *SF3B1*. The role of mutated EIF1AX was tested using loss of function approaches including viability and translational efficiency assays. Knockdown of both wild type and mutant EIF1AX was lethal to uveal melanoma cells. We probed the function of N-terminal tail EIF1AX mutations by performing RNA sequencing of polysome-associated transcripts in cells expressing endogenous wild type or mutant EIF1AX. Ribosome occupancy of the global translational apparatus was sensitive to suppression of wild type but not mutant EIF1AX. Together, these studies suggest that cells expressing mutant EIF1AX may exhibit aberrant translational regulation, which may provide clonal selective advantage in the subset of uveal melanoma that harbors this mutation.

## Introduction

Uveal melanoma (UM), which accounts for 5% of all melanomas, occurs in the iris, ciliary body, and choroid of the eye. Approximately 50% of UM patients develop metastatic disease, most often to the liver [1]. Primary UM is treated by either enucleation or radiation, while metastatic UM has no effective therapies and a median survival rate of approximately 6 months [2,3]. Thus, improved treatment of metastatic UM represents an unmet medical need.

Over 80% of UM tumors harbor activating hotspot mutations in *GNAQ* or *GNA11*, which encode alpha subunits of guanine nucleotide binding (G) proteins [4,5]. Mutations at residues 183 and 209 of these proteins result in constitutive downstream signaling to the protein kinase C, mitogen-activated protein kinase (MAPK), and YAP1 pathways [6–9]. Frequently observed copy number alterations in UM tumors include loss of a single copy of chromosome 3 (monosomy 3), amplification of 8q or 6p, and less frequently 8p gain or 1p, 6q, 16q loss [10,11]. Monosomy 3 is predictive of worse prognosis [12] and often co-occurs with loss of function mutations in the tumor suppressor BAP1, which is located on chromosome 3 [13]. In addition to *GNAQ*, *GNA11*, and *BAP1*, recurrent mutations in the splicing factor, *SF3B1*, as well as the translation initiation factor, *EIF1AX*, have been recently characterized in primary UM tumors [14,15].

Despite these advances, large-scale genome characterization efforts in UM have been limited by sample size and largely restricted to primary tumors. To expand knowledge of the somatic genetics of primary and metastatic UM, we sequenced the protein-coding exons of 52 primary tumors and 3 liver metastases derived from 2 patients. We also performed systematic functional studies focused on EIF1AX, a translation factor recurrently mutated in UM.

## Results

### Somatic mutations in primary uveal melanoma

**Fig 1:**
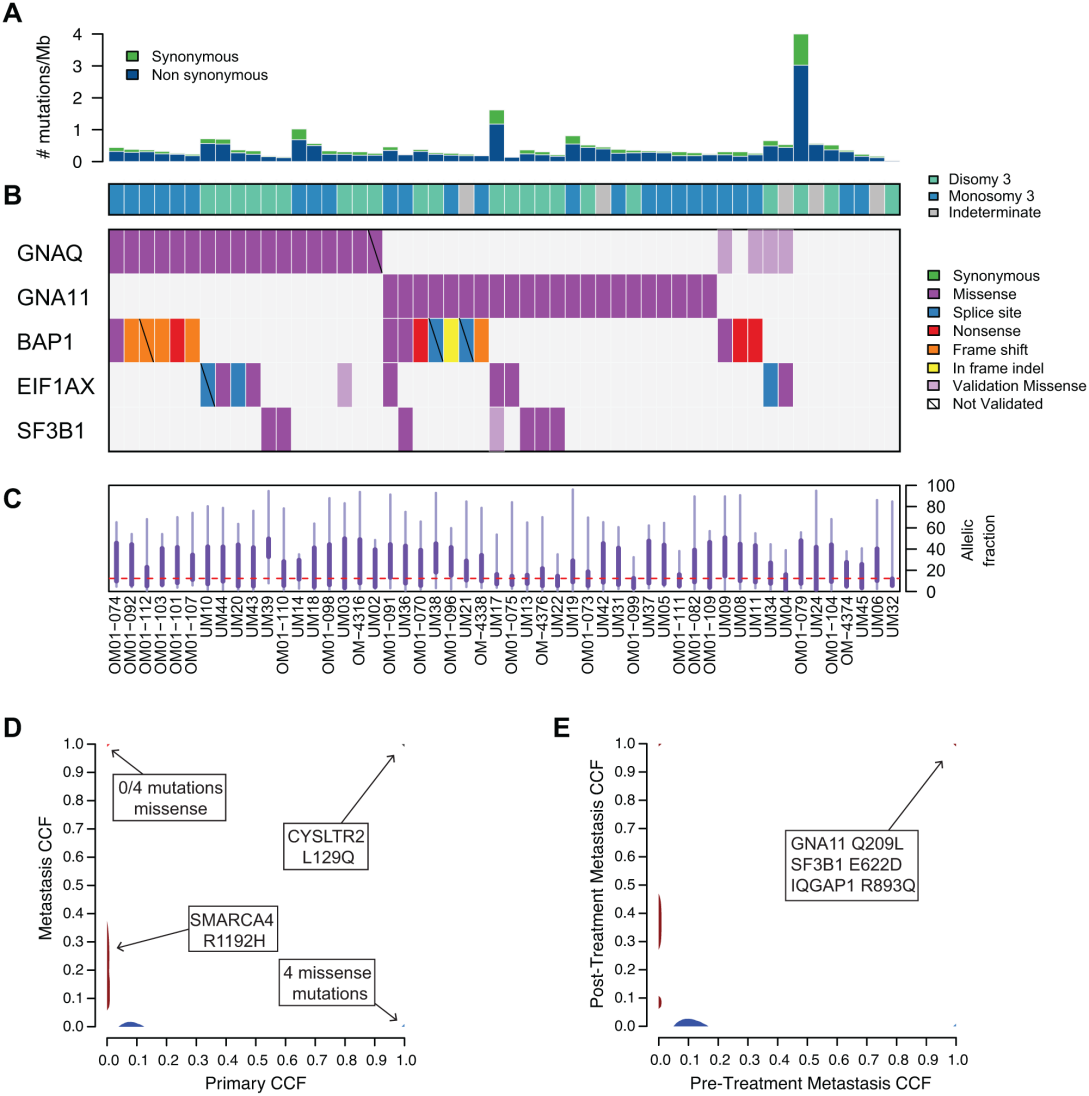
Somatic mutations in primary and metastatic uveal melanoma. **(A)** The number of synonymous and nonsynonymous mutations per megabase of DNA sequence for 52 samples, arranged in columns. **(B)** Mutations in recurrently mutated genes are color-coded and ordered by significance. **(C)** Boxplots represent the distributions of allelic fractions observed per sample where the thick line represents 25-75^th^ percentile, and thin line 5-95^th^. **(D)** The percentage of tumor cells (CCF) harboring a given mutation in the primary tumor in comparison to a metastatic liver sample from the same patient (UM45). **(E)** As in (D), but comparing a pre-treatment metastatic tumor sample to a post-treatment metastasis (Trio 2).

Solution-phase hybrid capture and whole exome sequencing (WES) were performed on paired primary tumor and normal genomic DNA from 61 patients with uveal melanoma (UM). Tumors were derived from enucleations and represent more advanced stages (Table 1). 101-fold mean target coverage was achieved, with an average of 90% of exonic bases covered per sample (S1 Table). A subset of 52 tumor/normal pairs passed standard quality control metrics, including screening for tumor DNA in normal samples or normal DNA in tumor samples (Table 1; S2 Table). The mean somatic mutation rate was 0.46 mutations per megabase (range: 0.031 to 4.00), with an average of 32 coding mutations per patient (Fig 1A; S1 Table). As expected [15,16], and in contrast to cutaneous melanoma [17], no signature of ultraviolet (UV) light-induced mutational damage was observed in this UM cohort.

**Table 1:** Clinical characteristics of analysis set.

Patients	MEEI (N = 23)	MDACC (N = 29)
Age at diagnosis (Median; range)	65 (34–89)	62 (23–83)
Normal DNA source	Blood	Adjacent choroid
**Gender**		
Male	14	17
Female	9	12
**Anatomic Site**		
Choroid	10	22
Choroid/CB	12	6
CB/Iris	1	1
**Tumor Stage**		
I	0	2
II	4	14
III	17	12
IV	0	1
N/A	2	0

Chromosomal copy number profiles were also generated using the CapSeg algorithm. Monosomy at chromosome 3 was observed in 53% of patients (S1 Fig). Consistent with prior studies [12], the majority of patients who had or went on to develop metastatic disease (11/13) harbored monosomy 3 (S2 Table). This observation was further validated by Kaplan Meier analysis, which indicates the expected correlation between monosomy 3 and overall survival reflecting death from metastatic uveal melanoma or any cause (S2A and S2B Fig). Consistent with prior analyses, presence of BAP1 mutation, versus either a SF3B1 or EIF1AX mutation portends a poorer survival (S2C and S2D Fig) [13–16]. As expected [18,19], chromosome 8q copy gains tended to co-occur with monosomy 3, whereas chromosome 6p copy number gains did not in this cohort. Other frequent chromosomal changes included chromosome 8p gains and 1p, 6q, 16q deletions.

To identify significantly mutated genes in UM, we used the MutSigCV algorithm, which accounts for patient and gene-specific mutation frequency, gene size, and the ratio of synonymous to nonsynonymous mutations per gene [17]. Four genes (*GNAQ*, *GNA11*, *BAP1*, and *EIF1AX*) were mutated more frequently than expected by chance (Fig 1B). All of these genes have been previously implicated in UM [4,5,13,15]. We observed 6 mutations in *SF3B1* at residues 625, 662, and 666. Although *SF3B1* did not meet the significance threshold in this cohort, it is a known cancer gene that is also recurrently mutated in UM [14]. Allelic fraction analysis of called mutations was used to confirm that mutations were present at detectable levels (Fig 1C). All somatic mutations identified in this cohort are available in Supplementary Material (S3 Table).

Targeted re-sequencing in all 52 tumor/normal pairs independently validated the somatic mutations identified in these 5 genes. In total, 98.4% of missense/nonsense mutations and 42.9% of small insertions/deletions were validated (S4 Table). Re-sequencing also identified several additional hotspot mutations in *GNAQ* (Q209), *EIF1AX* (G15), and *SF3B1* (R625) (Fig 1B). Most of these mutations occurred in regions of low WES sequence coverage, which may explain why they were not called initially by standard analysis algorithms.

The majority of patients harbored mutually exclusive mutations at residues 209 or 183 of *GNAQ* and *GNA11* (Fig 1B). One sample (OM-01-110) contained a novel *GNAQ* mutation, which harbored two mutations at the same codon (GGA>CTA) resulting in a G48L substitution. Sequencing reads spanning codon 48 confirmed that these mutations occur in cis. Residue 48 lies within a putative GTP-binding region of GNAQ (http://www.uniprot.org), suggesting a potential functional role, although its actual effect is unknown.

Mutations in *BAP1*, *EIF1AX*, and *SF3B1* were almost entirely mutually exclusive of each other (although most often co-occurring with *GNAQ* and *GNA11* mutations), with only 2 out of 29 mutant samples harboring alterations in more than one gene. This observation suggests three predominant genetic classes of UM, defined by these three recurring mutations, and is supported by data indicating that mutant *BAP1* is associated with a worse prognosis, while *EIF1AX* and *SF3B1* mutations indicate a better prognosis [13–15]. Indeed, mutations in EIF1AX occurred more frequently in Disomy 3 patients (S1 Fig), consistent with prior findings [15]. No patients with EIF1AX or SF3B1 mutations in this cohort are known to have developed metastatic disease with the exception of 2 patients that had co-occurring BAP1 mutations (S2 Table). Patient OM-091 harbored missense mutations in both *EIF1AX* (R13C) and *BAP1* (N102K); however, mutant *EIF1AX* was present at a low allelic fraction (0.051), suggesting a subclonal event. Sample UM36 contained missense mutations in both *BAP1* (G185R; with an allelic fraction of 0.83) and *SF3B1* (K666T; with an allelic fraction of 0.48). The majority of *SF3B1* mutations in UM occur at residue 625, however, lysine 666 is recurrently mutated in CLL [20]. Both OM-091 and UM36 patients died of metastatic UM (S2 Table), consistent with previous studies linking *BAP1* mutations in primary UM with a high likelihood of developing metastatic disease [13].

### Somatic mutations in metastatic uveal melanoma

To search for mutated genes in the metastatic setting, we also sequenced tumors from two UM patients with metastatic disease (UM45 and Trio 2). We utilized the ABSOLUTE algorithm to assign each somatic mutation a cancer cell fraction (CCF), which corresponds to the percentage of tumor cells harboring the genetic event [21]. We then utilized these cancer cell fractions to identify and compare clonal and subclonal events across distinct tumor samples from the same patient [22].

For patient UM45, the paired primary enucleated tumor and a single metastatic sample from a liver biopsy were sequenced. Only 4 clonal missense mutations were present in both samples (*MAN2A1*, *UHRF1BP1L*, *HCFC2*, *CYSLTR2*), including the recently described L129Q alteration in CYSLTR2 [23] (Fig 1D, top right). Phylogenetic analysis using CCFs suggests these tumors are siblings, which indicates that both the primary and metastatic cells evolved from a common initial tumor. In this patient, *SMARCA4*^*R1192H*^ was enriched in the metastatic sample compared to the primary tumor (Fig 1D; primary CCF 0, metastatic CCF 0.12). This observation was further validated using targeted resequencing (S3A Fig). SMARCA4 (or BRG1) is a catalytic subunit of the SWI/SNF chromatin remodeling complex, which undergoes somatic mutation in multiple cancers [24]. Although the functional consequence of this mutation within the cellular context of uveal melanoma awaits further study, codon 1192 resides within the conserved C-terminal helicase domain and is recurrently mutated across multiple cancer types (http://www.cbioportal.org).

For Trio 2, pre- and post-treatment liver metastases were sequenced, in addition to a normal sample (no primary tumor sample was available in this case; see S3B Fig for a clinical timeline). Therapies included an HSP90 inhibitor, a temozolamide/sorafenib combination, and a MEK inhibitor. Here, the pre-treatment tumor harbored only 4 missense mutations that were not observed in the post-treatment sample (1 clonal and 3 subclonal), while the post-treatment sample harbored 25 mutations that were not observed in the pre-treatment sample (1 clonal and 24 subclonal). Phylogenetic analysis suggests that these samples are siblings, indicating independent evolution from the same tumor. Seven clonal missense mutations were present in both samples, including *GNA11*^*Q209L*^, *SF3B1*^*E622D*^, and *IQGAP1*^*R893Q*^ (Fig 1E; S5 Table). *IQGAP1* encodes Ras GTPase-activating-like protein, however, it does not harbor Ras-GAP activity and instead functions as a scaffold protein in several signaling pathways that contribute to tumorigenic phenotypes including invasion and metastasis [25]. *IQGAP1* is not significantly mutated in any cancer type [26], but rather is frequently overexpressed [25]. All somatic mutations and CCF values determined by ABSOLUTE are available in Supplementary Material (S5 Table).

### Mutant and wild type EIF1AX play essential roles in uveal melanoma cells

**Fig 2:**
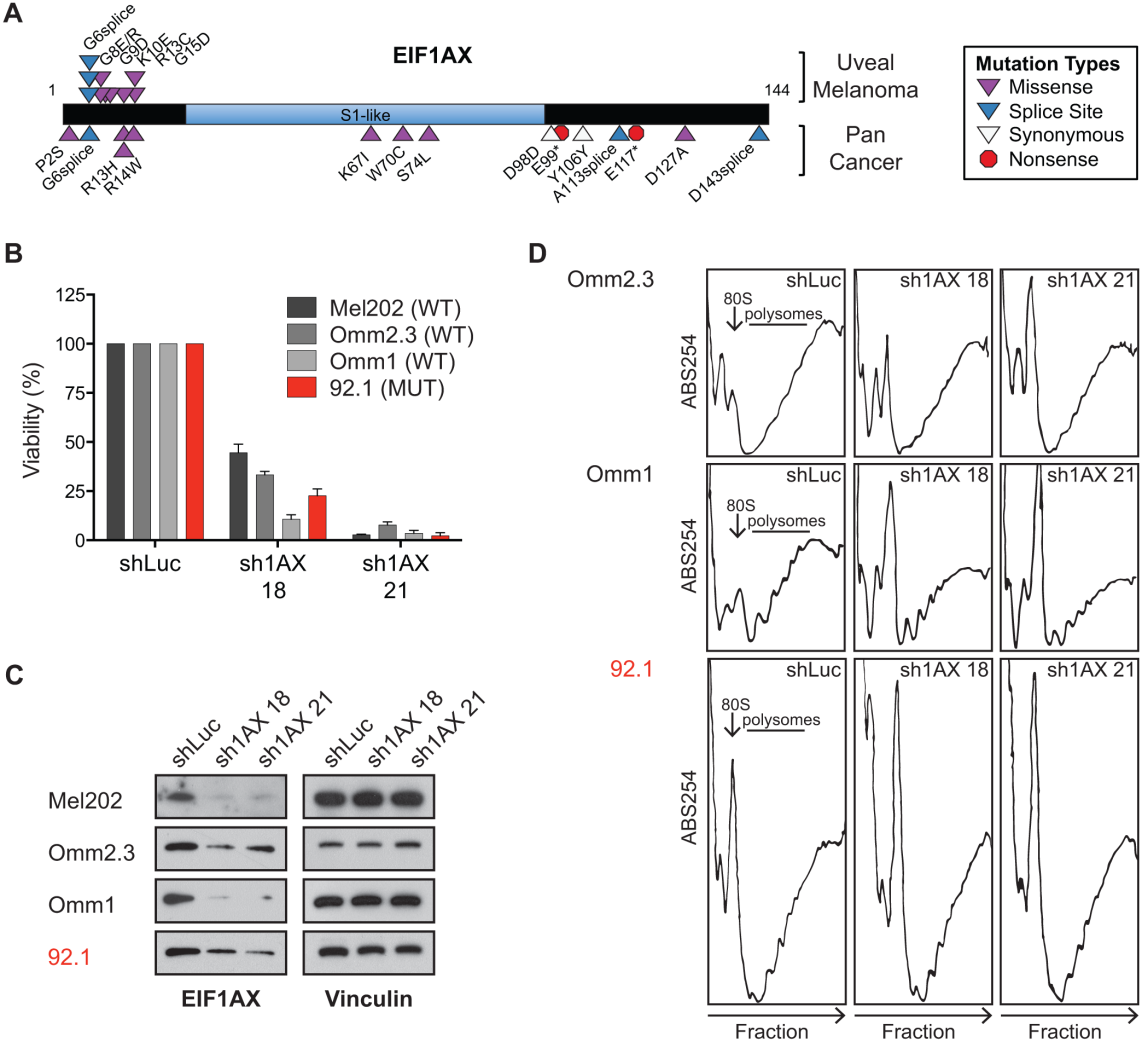
EIF1AX-regulated growth and translation in uveal melanoma. **(A)** Distribution of *EIF1AX* mutations observed in cohort of 52 uveal melanomas in comparison to other cancer types (as reported by http://www.tumorportal.org). **(B)** *EIF1AX* wild type (WT) or mutant (MUT) uveal melanoma cells were infected with *EIF1AX* or control shRNAs and cell viability was determined after 6 days using MTS. Percent growth is relative to shLuc-expressing cells. Error bars represent SD of mean from 3 independent experiments. **(C)** Immunoblot analysis of EIF1AX protein levels in shRNA-expressing cells. **(D)** Polysome profiles of cell lines expressing shRNAs against *EIF1AX* and *Luciferase*.

The x-linked translation initiation factor, *EIF1AX*, is the most recently described significantly mutated gene in UM and has not been functionally characterized in cancer. Consistent with prior studies [15], we observed recurrent mutations within the N-terminal 15 amino acids of *EIF1AX* that co-occur with GNAQ or GNA11 mutations (Fig 2A). This UM clustering pattern stands in contrast to pan-cancer analyses in which synonymous and non-synonymous mutations were observed throughout the *EIF1AX* coding region [26], however, in specific cancer types such as papillary thyroid carcinoma, recurrent mutations localized to the N-terminal tail (NTT) were observed [27]. All *EIF1AX* mutations in our UM cohort were non-synonymous and localized within the unstructured N-terminal tail (NTT) [28]. In addition, no inactivating mutations such as nonsense or frameshift mutations were observed. Together, these observations raise the possibility of a gain-of-function mutational event.

We also identified a homozygous G→A mutation at the start of exon 2 in a single established UM cell line (92.1) (S4 Fig). Transcriptome analysis (RNAseq) of 92.1 cells demonstrated faithful transcription of the G6D missense mutation, without evidence for altered mRNA splicing or wild type allele expression (S6A Fig). This finding is consistent with prior studies demonstrating exclusive expression of mutant *EIF1AX* mRNA in tumor samples from both male and female individuals [15]. These data suggest that in female patients, the wildtype allele is located on the inactivated X chromosome. Also consistent with prior studies [15], the majority of *EIF1AX*-mutant tumors in this cohort were male (7/10). However, the 92.1 cell line containing a homozygous mutation was from a female patient, suggesting poor sequencing of the inactivated X chromosome or genetic loss of the *EIF1AX* region. Examination of even larger cohorts may provide further insight into association of *EIF1AX* mutations and sex chromosome inactivation.

*EIF1AX* encodes an essential component of translation initiation [29]. Binding of EIF1AX to the small ribosomal subunit aids in assembly of the pre-initiation complex (PIC) and facilitates mRNA scanning [30–32]. More specifically, the EIF1AX-NTT stimulates PIC formation, interacts with eIF2 and eIF3, and aids in identifying the initiator codon [33–35]. Therefore, somatic mutations in the *EIF1AX*-NTT may result in changes in translational regulation—either global or specific mRNA effects.

To gain preliminary insights into the function of mutant EIF1AX in UM cells, we used lentiviral shRNA knockdown to suppress EIF1AX expression in a panel of UM cell lines, including the 92.1 line, which contains an *EIF1AX*^*G6D*^ mutation. Expression of *EIF1AX* shRNAs both decreased *EIF1AX* gene expression and impaired UM cell viability (Fig 2B and 2C). Notably, both *EIF1AX*-mutant and *EIF1AX*-wild type UM cell lines exhibited suppressed viability following knockdown, suggesting that EIF1AX may be uniformly essential in UM cells. Based on these knock down results, *EIF1AX*-NTT mutations in UM likely do not solely confer loss of wild type protein function.

In light of these results, we sought to determine if *EIF1AX* might represent a dependency across multiple contexts. To assess this, we leveraged Project Achilles dataset 2.4.3 (http://www.broadinstitute.org/achilles), which consists of large-scale pooled shRNA screening (>90,000 shRNAs) across 216 cancer cell lines of various lineages [36]. In Project Achilles, cells were infected with a pool of shRNAs and the relative levels of shRNAs present at 16 population doublings was compared to the initial plasmid pool. Those shRNAs selectively depleted after 16 population doublings may indicate genes that are essential to the viability of the relevant cell lines.

Five shRNAs targeting *EIF1AX* were present in the Project Achilles dataset. Of these, two were strongly depleted across all 216 cell lines (S5A Fig, top). Although off-target effects cannot be entirely ruled out, these 2 shRNAs were also the most effective at suppressing EIF1AX protein levels (S5B Fig). The magnitude of shRNA depletion was similar to the gene that encodes ribosomal protein S6 (*RPS6*), a known essential gene (S5A Fig, bottom). Taken together, these data are consistent with EIF1AX being universally essential in eukaryotic cells, and suggest that cancer cells retain this dependency for pre-initiation complex assembly, growth, and survival, although further functional validation in each cancer type is necessary.

### Mutant EIF1AX regulated translation

We next sought to test the hypothesis that EIF1AX-NTT mutations might regulate translation of a distinct set of transcripts compared to wild type EIF1AX. To investigate this possibility, we performed polysome profiling of *EIF1AX* mutant and wild type UM cell lines expressing shRNAs targeting *EIF1AX* or *Luciferase* (shLuc; control). Interestingly, the 80S peak in the *EIF1AX* mutant cell line (92.1) showed greater amplitude than that seen in *EIF1AX* wild type cells (Omm2.3 and Omm1), raising the possibility that mutant EIF1AX might be associated with altered protein translation (Fig 2D). RNAi-mediated suppression of EIF1AX expression also augmented the 80S peak in all contexts tested, suggestive of reduced polysome formation and impaired translation initiation (Fig 2D).

EIF1AX-regulated translation was further probed by massively parallel sequencing of total mRNA and mRNAs from polysome fractions. Sequencing of polysome-associated transcripts provides a means to identify those mRNAs that may undergo active translation in the cells. The ratio of polysome-associated mRNA levels to total mRNA levels from input lysate can be used to calculate the “translational efficiency” of each transcript [37]. RNAseq was used to confirm extent of EIF1AX knockdown in shRNA expressing cells (S6B and S6C Fig).

To identify transcripts regulated by EIF1AX, we tested which polysomal/total RNA ratios were significantly altered upon EIF1AX knockdown. Here, the expression levels for each gene were first normalized using the trimmed mean of M values (TMM) method [38]. This approach assumes that most genes are not differentially expressed between samples and identifies a sample-specific scaling factor to allow for more accurate comparisons. Next, we identified genes whose translational efficiency was affected by the knockdown of EIF1AX.

By this method, 896 genes achieved significance (p-value < 0.05), but only 142 were deemed to be sufficiently expressed for more detailed analysis (based on whether 50% of the samples achieved a counts per million (CPM) of > 2 in the RNAseq data; S6 Table). Of these, nearly all showed reduced translational efficiency—only 7 genes showed increased translational efficiency following EIF1AX knockdown. These data support the function of EIF1AX as an initiation factor essential for translation and raise the possibility that certain genes are particularly susceptible to EIF1AX depletion.

**Fig 3:**
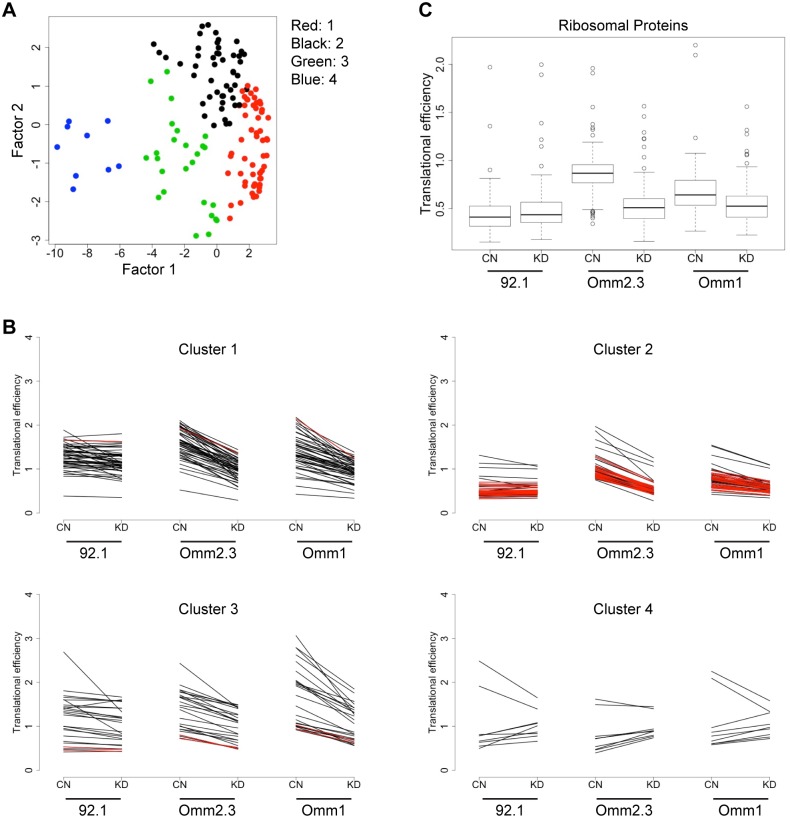
Decreased EIF1AX expression impairs translation of protein synthesis machinery in wildtype, but not mutated setting. **(A)** Principal component analysis depicts 4 color-coded clusters of 141 genes. **(B)** The trend in translational efficiency is depicted for each cluster in cells expressing control shRNAs (CN) or *EIF1AX* shRNAs (KD). Each line represents a different gene. Ribosomal protein genes are highlighted in red. Translational efficiency was calculated as polysome CPM / total CPM. **(C)** Boxplots demonstrate the distribution of the translational efficiencies of 78 ribosomal proteins in cells as in (B).

Next, we categorized the genes based on their translational efficiency profiles. The 142 expressed genes that showed differential translational efficiency were grouped into 4 clusters based on translational efficiency using k-means clustering (Fig 3A). Overall, the genes within a cluster display a common change in translational efficiency following EIF1AX knockdown (Fig 3B). Several genes in cluster 1 demonstrate decreased translational efficiencies across all three cell lines (*C20orf24*, *GET4*, *GFER*, *PDF*, *SAC3D1*, and *TMEM160)*. These genes represent those commonly regulated by wild type and mutant EIF1AX. In contrast, cluster 2 genes show reduced translational efficiencies in the wild type cell lines (Omm2.3 and Omm1), but no change in the *EIF1AX*-mutant cell line (92.1). This cluster includes 26 ribosomal protein genes (Fig 3B, red). These data indicate that knockdown of wild type EIF1AX results in significantly suppressed translation of ribosomal proteins, while knockdown of mutant EIF1AX levels does not.

We were intrigued by the observation that one of the clusters we identified was enriched for ribosomal protein genes. Translation of ribosomal protein genes is regulated in a coordinated manner by a 5’ terminal oligopyrimidine tract (5’ TOP) within the mRNA sequence [39]. We found that the median translational efficiency of all ribosomal protein genes was reduced following EIF1AX knockdown in wild type, but not mutant cells (Fig 3C). This was the only gene family that exhibited extensive coordinated regulation in the context of EIF1AX knockdown. In addition, these genes demonstrated already reduced translational efficiency in the mutant cell line (92.1), and were not additionally affected by EIF1AX knockdown in this setting (Fig 3C). Together, these data raise the possibility that wild type EIF1AX may regulate translation of ribosomal protein genes, but that this regulation may be diminished in at least some *EIF1AX*-mutant settings.

## Discussion

In this study, we sequenced the exomes of 52 UM primary tumor/normal pairs. To our knowledge, this represents one of the largest UM cohorts to undergo comprehensive genomic characterization to date. Consistent with prior studies, recurrent mutations were observed in *GNAQ*, *GNA11*, *BAP1*, *EIF1AX*, and *SF3B1* [4,5,13–15]; no additional genes met the threshold for significance. Based on the observation that the known recurrently mutated gene, SF3B1, did not reach significance even though 6 mutations were observed by WES, there may have been other mutations that were also missed. This study confirms the relative mutually exclusive nature of *BAP1*, *SF3B1*, and *EIF1AX* gene mutations. Our data further confirm [15,16] that in contrast to cutaneous melanoma, which has the highest somatic gene mutation frequency of any cancer type [17], UM lacks a UV-radiation mutation signature and has a low mean somatic mutation rate. Recent data suggest that clinical benefit to immune checkpoint blockade therapy (e.g., anti-CTLA4) in cutaneous melanoma is associated with higher mutational burden [40]. A higher mutation frequency is thought to generate a diversity of neoantigens that can be recognized by the immune system. Thus, the low somatic gene mutation frequency observed in this and other UM cohorts may explain the lower response rates thus far reported for UM tumors using the recently FDA-approved immune checkpoint blockade inhibitors in cutaneous melanoma [41,42].

In two cases, we sequenced multiple tumors from the same patient. This allowed us to track enrichment or depletion of specific somatic alterations over the course of disease progression. To date, monosomy 3 and mutated *BAP1* have been associated with UM metastasis [12,13], but limited analyses of metastatic tumor genomes have been reported. In UM45 in this cohort, a *SMARCA4* mutation was detected in the metastatic sample. *SMARCA4* mutations were recently reported in metastatic UM tumors, although matched primary tumor DNA was not sequenced for comparison [43]. Conceivably, then, SMARCA4 may contribute to the establishment or maintenance of metastatic disease, although functional studies are needed to confirm this hypothesis. Some *SMARCA4*-mutant cancer cells may be sensitive to inhibition of the second SWI/SNF catalytic subunit [44–46]. If SWI/SNF mutations are recurrently linked to metastatic disease in UM, the relevance of this synthetic lethal relationship to residual SWI/SNF complex function may merit additional exploration.

We identified *EIF1AX* N-terminal tail mutations in approximately 20% of primary UM tumors, consistent with prior studies [15]. Putative driver mutations in this translation factor are of interest given that increased protein synthesis is frequently observed in rapidly proliferating cancer cells. Both upstream signaling pathways and aberrant translation factor expression contribute to this phenomenon [47]. Indeed, several translation initiation factors that are overexpressed in human cancer, including EIF4E, EIF4G, EIF3A, EIF3C, and EIF3H may also contribute to tumorigenesis [48]. Consistent with these general observations, our RNAi knockdown experiments suggest an essential role for EIF1AX in both wild type and mutant settings. *EIF1AX* is the first translation initiation factor reported as recurrently mutated in cancer, and is significantly mutated in both UM and papillary thyroid carcinoma [27].

Recent ribosome footprinting experiments demonstrate that translation of the protein synthesis machinery is suppressed by pharmacologic inhibition of mTOR, a well-known regulator of translation [49,50]. We performed RNA sequencing of polysome-associated mRNAs and observed similar suppression of the translational efficiency of ribosomal proteins following EIF1AX knockdown in wild type *EIF1AX* cells. In contrast, mutant *EIF1AX* cells did not display this phenotype. There are at least two possible explanations for these observations. First, the level of knockdown achieved may not be sufficient to impair mutant EIF1AX function (thus, ribosomal proteins continue to be translated sufficiently by this mechanism). This model could be operant if mutated EIF1AX has enhanced function over wild type. However, the observed 80S accumulation following shRNA expression is consistent with the notion that the magnitude of EIF1AX knockdown achieved here is functionally consequential. Second, translation in the setting of mutant EIF1AX may be less efficient overall, such that knockdown does not further impair ribosomal functions. This model is supported by the higher 80S peak observed in mutant EIF1AX cells in comparison to wildtype (Fig 2D). This model may also imply that mutant EIF1AX harbors previously unrecognized yet essential functions that may or may not be related to translation. For example, one could posit a neomorphic role for EIF1AX, resulting in regulation of a unique set of mRNAs or a translation-independent activity contributing to clonal advantage. However, no evidence for such a neomorphic effect currently exists. As *EIF1AX* mutations are associated with a low risk of developing metastatic disease, additional functional studies are required to determine the specific mechanisms by which *EIF1AX* mutations provide a selective advantage to UM cells in which they are present.

In summary, this study utilized systematic genomic approaches to probe the somatic genetics of primary and metastatic UM, as well as the function of mutated EIF1AX. This is the first study of deregulated translation by a mutated initiation factor in cancer. Future work should focus on further developing approaches to target the described genetic lesions, in particular in patients with metastatic potential.

## Materials and methods

### Tumor specimens

This study was approved by the institutional review boards of the Massachusetts Eye and Ear Infirmary, MD Anderson Cancer Center, Dana-Farber Cancer Institute, and the Broad Institute. All patients provided written informed consent. Biopsy samples were obtained from 62 patients with UM. Primary tumor samples were from enucleated eye specimens, while metastatic samples were from core biopsies obtained by interventional radiology. Fresh tissue was immediately flash frozen at -80°C.

### DNA extraction and whole exome sequencing

Germline DNA was isolated from peripheral blood (N = 26) or adjacent normal choroidal tissue (N = 35). Detailed sequencing and analysis methods are in Supplementary Material (S1 File). Sequencing data will be deposited in dbGaP (http://www.ncbi.nlm.nih.gov/gap) (accession # phs001370.v1.p1).

### Cell lines

92.1, Mel202, Mel270, Omm2.3, Omm2.5, Omm1, and Mel285 were kindly provided by Martine Jager in 2011 (Leiden University, The Netherlands). The origins of these cell lines were previously described [51]. Cells were maintained in RPMI-1640 with 10% heat-inactivated FBS and identity confirmed by fingerprinting using the GenePrint 10 kit (Promega) in January 2014. An *EIF1AX* mutation in exon 1 or 2 was observed in only the 92.1 cell line of the 7 lines tested.

### Lentiviral infections of shRNAs

shRNA expression constructs were obtained from The RNAi Consortium of the Broad Institute in the lentiviral expression plasmid pLKO.1 and include:

shLuc (TRCN0000072243, 5’-CTTCGAAATGTCCGTTCGGTT-‘3)
sh1AX 18 (TRCN0000062618, 5’-CCGAGACTACCAGGATAACAA-3’)
sh1AX 21 (TRCN0000062621, 5’-CCTGGAGATGATGATGAAATT-3’)

Lentivirus was produced by transfecting 2.4e6 293T cells with 3μg pLKO.1, 2.7μg Δ8.9 (*gag*, *pol*), and 0.3μg VSV-G plasmids in the presence of 18μL Xtreme Gene 9 (Roche). Virus was harvested 72h after transfection and filtered using a 0.45μm syringe. For shRNA expression, cells were seeded at 2 to 4 x 10^5^ cells/well in 6-well plates for immunoblot assays and polysome profiling and 1 to 2 x 10^3^ cells/well in 96-well plates for cell viability assays. Cells were infected 24h later (1:40–1:50) in the presence of polybrene (Millipore; 5μg/mL) and then centrifuged at 2,250 RPM for 30 minutes at 37°C followed by a media change. To confirm infection efficiency, puromycin (1μg/mL) was added 24h after infection to half the 96-well plate. For polysome profiling, cells were passaged into 10cm dishes 24h prior to harvesting.

### Viability assays

Cells were infected in 96-well format (as described). Fresh media was added 72h after shRNA infection and proliferation was assessed in triplicate after another 72h (6 days total) using CellTiter96 Aqueous assay (Promega). Viability was calculated as a percentage of the control (shLuc) after background subtraction.

### Immunoblot analysis

Cells were washed twice with cold PBS and lysed in RIPA buffer containing Halt protease and phosphatase inhibitor single-use cocktail (Pierce). Lysates were quantified using Bradford assay (Bio-Rad), resolved by SDS gel electrophoresis (Invitrogen), transferred to nitrocellulose (Invitrogen) or PVDF (Millipore) membranes, and then blocked with 5% milk and probed with primary antibodies for EIF1AX (Pierce 1:1,000) and Vinculin (Calbiochem 1:5,000). HRP-linked secondary antibodies (anti-rabbit, anti-mouse IgG; 1:2,000 dilution, Santa Cruz) followed by chemiluminescence (Pierce) were used for detection.

### Polysome fractionation and RNA isolation

Sub-confluent cells were treated with 100μg/mL cycloheximide for 3 minutes at 37°C and then washed twice with cold PBS containing 100μg/mL cycloheximide. Cells were lysed in buffer (5mM Tris (pH7.4), 2.5mM MgCl_2_, 1.5mM KCl, 2mM DTT, 0.5% Triton-X, 0.5% NaDOC, 0.1units/μL RNasin, 100μg/mL cycloheximide, and 1x Halt protease and phosphatase inhibitor single-use cocktail), and then incubated on ice (20 min) and centrifuged at 13,000 RPM (10 min; 4°C). Lysates were quantified using Abs260 and equal amounts were loaded onto 12mL 10%-50% sucrose gradients (15mM Tris (pH7.4), 15mM MgCl_2_, 150mM NaCl, 100μg/mL cycloheximide). Gradients were centrifuged at 35,000 RPM for 2 hours at 4°C (SW40Ti rotor) and then 1mL fractionated with Auto Densi-Flow connected to RediFrac with monitoring of absorbance at 254nm. RNA was extracted from input lysate and polysome fractions using Trizol LS (Invitrogen) and standard phenol/chloroform extraction methods followed by an isopropanol precipitation. Pellets from polysome fractions were pooled and an additional ethanol precipitation was performed.

### RNA sequencing and analysis

The Quant-iT RiboGreen RNA Assay Kit (Invitrogen) was used to quantify RNA. cDNA library construction was performed using the TruSeq RNA Sample Preparation protocol (Illumina, Revision A, 2010). Additional details are provided in Supplementary Material (S1 File). For analysis, read counts for each gene were used to normalize the data using the TMM method [38]. Genes whose translation efficiency was affected by the knockdown of EIF1AX were then identified using the edgeR package [52]. To this end, we fitted the following generalized linear model, based on a negative binomial distribution, after estimating the expression level-detrended and gene-wise overdispersion.

$$count_{i}\;\sim \;\alpha_{i}*cell\_line+\beta_{i}*replicate+\;\gamma_{i}*knockdown+\;\delta_{i}*fraction+\vartheta_{i}*knockdown*fraction+\varepsilon_{i}$$

where:

count_i_ is the normalized gene counts for gene i

cell line is the vector representing the cell line effect (levels = 92.1, Omm2.3, Omm1)

replicate is the vector representing the replicate experiments (two independent replicate experiments performed)

fraction is the vector representing the profiling of total or polysomal RNA

The interaction effect ζ_i_*knockdown*fraction models genes with transcripts that display a shift in the ratio between the polysomal and total RNA fraction (= translational efficiency) upon EIF1AX suppression. The genes of interest have a coefficient ζ_i_ significantly different from zero. Genes were nominated based on an interaction p-value threshold of 0.05 and an expression threshold of at least 50% of the samples containing a counts per million (CPM) greater than 2. Genes were then grouped by a k-means clustering of the translational efficiencies using correlation-based (Pearson correlation) similarity measures. One gene was removed due to division by 0 CPM (*XKR6*). Four clusters were identified and the trend in the translational efficiencies across control and knockdown samples for each cell line was depicted. Translational efficiencies were defined as polysome CPM / total CPM. The average translational efficiency for control and knockdown was computed and graphed using line plots.

## Supporting information

S1 File Supplementary materials and methods. (DOC)

S1 Table Sequencing metrics of 52 uveal melanomas. (XLS)

S2 Table Clinical characteristics of 52 uveal melanomas. (XLS)

S3 Table All somatic alterations identified by whole exome sequencing in 52 pairs. (XLS)

S4 Table Validation of selected mutations by targeted resequencing. (XLS)

S5 Table All somatic alterations identified in trio analysis. (XLS)

S6 Table Genes with significant changes in translational efficiency following EIF1AX knockdown. P-value and cluster is indicated for each gene.
(XLS)

S1 Fig Copy number profiles of 52 uveal melanomas. Coverage values from whole exome sequencing were converted into segmentation files and visualized using IGV. Samples are ordered by chromosome 3 status, or labeled Indeterminate (Ind.) due to noise across the genome. 1AX indicates samples with an *EIF1AX* mutation.
(PDF)

S2 Fig Kaplan-Meier analysis of recurrent alterations in cohort. **(A and B)** Kaplan-Meier analysis showing primary uveal melanoma patients with evaluable OS data who had either monosomy 3 (n = 20) or disomy 3 (n = 23) tumors. OS reflective of death from metastatic uveal melanoma; the median survival was 1216 days in the monosomy 3 cohort and not reached in the disomy 3 cohort. Log-rank p value = 0.0034. HR = 6.9, 95% Cl (1.7 to 15.5) **(B)** As in (A), but overall survival reflective of death from any cause; the median survival was 1008 days in the monosomy 3 cohort and not reached in the disomy 3 cohort. Log-rank p value = 0.0034. HR = 4.5, 95% Cl (1.6 to 10.3). **(C)** Kaplan-Meier analysis showing primary uveal melanoma patients with evaluable OS data with tumors harboring a BAP1 (n = 14) vs. SF3B1 or EIF1AX (n = 14) mutation. OS reflective of death from metastatic uveal melanoma; the median survival was 744 days in the BAP1 mutant cohort and not reached in the SF3B1/EIF1AX mutant cohort. The Log-rank p value = 0.0008. HR = 13.7, 95% Cl (3.0 to 62.9). **(D)** As in (C), but OS reflective of death from any cause; the median survival was 744 days in the BAP1 mutant cohort and not reached in the SF3B1/EIF1AX mutant cohort. The Log-rank p value = 0.0022. HR = 7.5, 95% Cl (2.1 to 28.8). Note, sample UM 36 had both BAP1 and SF3B1 mutations, but was analyzed as a BAP1 mutant sample.
(PDF)

S3 Fig Trio mutation validation and clinical timeline. **(A)** IGV screenshot of SMARCA4 mutation from exome sequencing and targeted validation of UM45. **(B)** Trio 2 biopsy and treatment are indicated across time course.
(PDF)

S4 Fig EIF1AX sequence and protein expression across UM cell lines. **(A)** Exon 2 sequencing trace displays putative *EIF1AX*^*G6D*^ mutation in 92.1 cell line. **(B)** Immunoblot analysis of EIF1AX protein levels.
(PDF)

S5 Fig EIF1AX dependency across 216 cell lines from Project Achilles. **(A)** Histograms represent shRNA level scores (normalized log fold change) for 5 *EIF1AX* (top) and 5 *RPS6* (bottom) shRNAs from 216 cell lines in Achilles v2.4.3. Lower values represent more depletion indicating more dependency. *EIF1AX* shRNAs used in this study are bolded. **(B)** Immunoblot analysis of EIF1AX protein levels in 2 uveal melanoma cell lines expressing indicated shRNAs.
(PDF)

S6 Fig *EIF1AX* sequence and expression levels from polysome profiling RNAseq. **(A)** IGV screenshot of *EIF1AX* exon 2 start indicates exclusive mRNA expression of the G6D variant in the 92.1 cell line. **(B)** Heatmap displays *EIF1AX* expression levels in RPKM for total and polysome-associated mRNA in 92.1 cells expressing indicated shRNAs. **(C)** As in (B), but for Omm2.3 and Omm1 *EIF1AX*-wild type cells.
(PDF)
