# Supplementary material for: Systematic genomic and translational efficiency studies of uveal melanoma
Source: PLoS One. 2017 Jun 8;12(6):e0178189. doi: 10.1371/journal.pone.0178189 (PMC5464544; doi:10.1371/journal.pone.0178189)
Supplement: S1 File — (DOC) [file pone.0178189.s001.doc]

**S1 File. Supplementary materials and methods.**

**DNA Extraction and Whole Exome Sequencing**

The blood samples were extracted using Gentra Puregene Blood kit (Qiagen) and the fresh frozen tissue samples were extracted using QIAamp DNA Mini kit (Qiagen) according to manufacturer’s protocol. For all samples except Trio 2, DNA libraries and whole exome sequencing data were generated as previously described . Tumor and normal DNA went through shearing, end repair, adenylation, and adapter ligation using barcoded adapters. Hybrid capture was performed on size-selected DNA using the SureSelect v2 Exome Target Enrichment System (Agilent). Resulting libraries were quantified by qPCR using the KAPA Biosystems Library Quantification Kit, then pooled and sequenced across multiple HiSeq flow cell lanes (Illumina). 101x mean target coverage was achieved. For trio 2, DNA libraries and sequencing data were generated as previously described , with the following modification: hybrid capture was performed using the SureSelect v2 Exome baits (Agilent).

**Sequence Data Processing and Quality Control**

Standard methods developed at the Broad were used for data processing and analysis, as previously described . Sequence data were aligned to hg19 and BAM files were generated using Picard (http://picard.sourceforge.net/), and then input into Firehose for analysis (http://www.broadinstitute.org/cancer/cga/Firehose). Tumor and normal pairs were confirmed using genotyping. Sample cross-contamination was estimated using ContEst and ranged from 0.1 to 8.2%. Copy number profiles were reviewed and nonaberrant tumor samples or aberrant normal samples were removed from the analysis set (N=8). One hypermutated sample was excluded.

**Somatic Mutation Calling and Significance Analysis**

Somatic base pair substitutions and small insertions or deletions were identified using MuTect and Indelocator (http://www.broadinstitute.org/cancer/cga/indelocator), respectively, and annotated using Oncotator (http://www.broadinstitute.org/cancer/cga/oncotator). DNA oxidation related artifacts were identified and filtered using a published method . The significance of mutated genes was determined using MutSigCV . Mutations were manually reviewed by visual inspection using IGV . For copy number analysis, segment files were generated using sequencing coverage data compared to a panel of normal samples. The circular binary segmentation algorithm was applied . The presence of disomy 3 or monosomy 3 was determined by manual review of CapSeg plots. Samples with noisy copy number data were labeled indeterminate.

**Overall Survival Analysis**

Kaplan-Meier survival analysis was used for OS determinations. The interval of time employed for these analyses was from primary uveal melanoma tumor pathological diagnosis to documented death from uveal melanoma metastasis or death from any cause. Determination of Log-rank test p-values, median survival and hazard ratios were performed using GraphPad Prism 6 software.

**Trio Analysis**

The percentage of tumor cells (CCF) harboring each genetic alteration was determined using the previously described algorithm ABSOLUTE . Clonal events were those with a cancer cell fraction (CCF) of close to 1, while all other events were subclonal. Phylogenetic trees were assembled after comparing the clonal and subclonal events in each sample from a given individual .

**Mutation Validation**

Targeted resequencing of select mutations was performed using microfluidic PCR (Fluidigm). 22 assays were designed to cover 37 targets with amplicons ranging from 149-164bp in size. The entire analysis set (51 pairs and 1 trio) was assayed using all primer sets across three 48.48 Access Array IFC chips (Fluidigm). Resulting amplicons were pooled and sequenced on MiSeq (Illumina) with 150 base paired-end reads. Resulting BAM files were manually reviewed at sites of interest using IGV . *GNAQ* (R183 and Q209), *GNA11* (Q209), *SF3B1* (R625), and *EIF1AX* (exon 2) hotspot mutations were assessed across all samples.

**Cell Line DNA Extraction and Sanger Sequencing**

Cell line genomic DNA was extracted using DNeasy Blood & Tissue Kit (Qiagen) according to manufacturer’s instructions. Exons 1 and 2 of *EIF1AX* were PCR amplified using AccuPrime *Pfx* SuperMix (Life Technologies). PCR products were purified using QIAquick PCR Purification Kit (Qiagen) and then Sanger sequenced. Traces were visualized using SeqMan Lasergene 8 software (DNASTAR). Primers (purchased from Integrated DNA Technologies) include:

Exon 1 forward (5’-GAAAAGCGACGCAAAGAGTC-3’)

Exon 1 reverse (5’-CTGGGTGACCTGCAATCTAC-3’)

Exon 2 forward (5’-GAAGGGTAGGGAGGTGATAATG-3’)

Exon 2 reverse (5’-AGGCTGAAGTGGAAGGACTG-3’)

**Project Achilles Analysis**

The shRNA level scores for *EIF1AX* and *RPS6* from Project Achilles v2.4.3 (216 cell lines) (http://www.broadinstitute.org/achilles/) were visualized using histograms in Gene-E (www.broadinstitute.org/cancer/software/GENE-E/).

**RNA Sequencing and Analysis**

mRNA was selected from total RNA by oligo dT beads, followed by heat/ion fragmentation and double-stranded cDNA synthesis.  End repair, adenylation, adapter ligation, and PCR enrichment were performed. Resulting libraries were quantified by qPCR using the KAPA Biosystems Library Quantification Kit for Illumina Sequencing Platforms and then pooled and sequenced using Illumina HiSeq to a depth of 45-50 million reads per sample. The standard Broad Picard Pipeline was used to generate BAM files.  Sequence data were aligned to hg19 using TopHat 1.4.1 .  Read counts and RPKM (reads per kilobase of transcript per million mapped reads) were determined using RNA-SeQC .

References

1. Fisher S, Barry A, Abreu J, Minie B, Nolan J, et al. (2011) A scalable, fully automated process for construction of sequence-ready human exome targeted capture libraries. Genome Biol 12: R1.

2. Wagle N, Berger MF, Davis MJ, Blumenstiel B, Defelice M, et al. (2012) High-throughput detection of actionable genomic alterations in clinical tumor samples by targeted, massively parallel sequencing. Cancer Discov 2: 82-93.

3. Stransky N, Egloff AM, Tward AD, Kostic AD, Cibulskis K, et al. (2011) The mutational landscape of head and neck squamous cell carcinoma. Science 333: 1157-1160.

4. Banerji S, Cibulskis K, Rangel-Escareno C, Brown KK, Carter SL, et al. (2012) Sequence analysis of mutations and translocations across breast cancer subtypes. Nature 486: 405-409.

5. Ojesina AI, Lichtenstein L, Freeman SS, Pedamallu CS, Imaz-Rosshandler I, et al. (2014) Landscape of genomic alterations in cervical carcinomas. Nature 506: 371-375.

6. Cibulskis K, McKenna A, Fennell T, Banks E, DePristo M, et al. (2011) ContEst: estimating cross-contamination of human samples in next-generation sequencing data. Bioinformatics 27: 2601-2602.

7. Cibulskis K, Lawrence MS, Carter SL, Sivachenko A, Jaffe D, et al. (2013) Sensitive detection of somatic point mutations in impure and heterogeneous cancer samples. Nature Biotechnology 31: 213-219.

8. Costello M, Pugh TJ, Fennell TJ, Stewart C, Lichtenstein L, et al. (2013) Discovery and characterization of artifactual mutations in deep coverage targeted capture sequencing data due to oxidative DNA damage during sample preparation. Nucleic Acids Res 41: e67.

9. Lawrence MS, Stojanov P, Polak P, Kryukov GV, Cibulskis K, et al. (2013) Mutational heterogeneity in cancer and the search for new cancer-associated genes. Nature 499: 214-218.

10. Robinson JT, Thorvaldsdottir H, Winckler W, Guttman M, Lander ES, et al. (2011) Integrative genomics viewer. Nat Biotechnol 29: 24-26.

11. Thorvaldsdottir H, Robinson JT, Mesirov JP (2013) Integrative Genomics Viewer (IGV): high-performance genomics data visualization and exploration. Brief Bioinform 14: 178-192.

12. Olshen AB, Venkatraman ES, Lucito R, Wigler M (2004) Circular binary segmentation for the analysis of array-based DNA copy number data. Biostatistics 5: 557-572.

13. Carter SL, Cibulskis K, Helman E, McKenna A, Shen H, et al. (2012) Absolute quantification of somatic DNA alterations in human cancer. Nat Biotechnol 30: 413-421.

14. Landau DA, Carter SL, Stojanov P, McKenna A, Stevenson K, et al. (2013) Evolution and impact of subclonal mutations in chronic lymphocytic leukemia. Cell 152: 714-726.

15. Brastianos PK, Carter SL, Santagata S, Cahill DP, Taylor-Weiner A, et al. (2015) Genomic Characterization of Brain Metastases Reveals Branched Evolution and Potential Therapeutic Targets. Cancer Discov 5: 1164-1177.

16. Trapnell C, Roberts A, Goff L, Pertea G, Kim D, et al. (2012) Differential gene and transcript expression analysis of RNA-seq experiments with TopHat and Cufflinks. Nat Protoc 7: 562-578.

17. DeLuca DS, Levin JZ, Sivachenko A, Fennell T, Nazaire MD, et al. (2012) RNA-SeQC: RNA-seq metrics for quality control and process optimization. Bioinformatics 28: 1530-1532.
