# Supplementary figures and images for: Systematic genomic and translational efficiency studies of uveal melanoma
Source: PLoS One. 2017 Jun 8;12(6):e0178189. doi: 10.1371/journal.pone.0178189 (PMC5464544; doi:10.1371/journal.pone.0178189)

Supp. Fig. S1

Chromosome

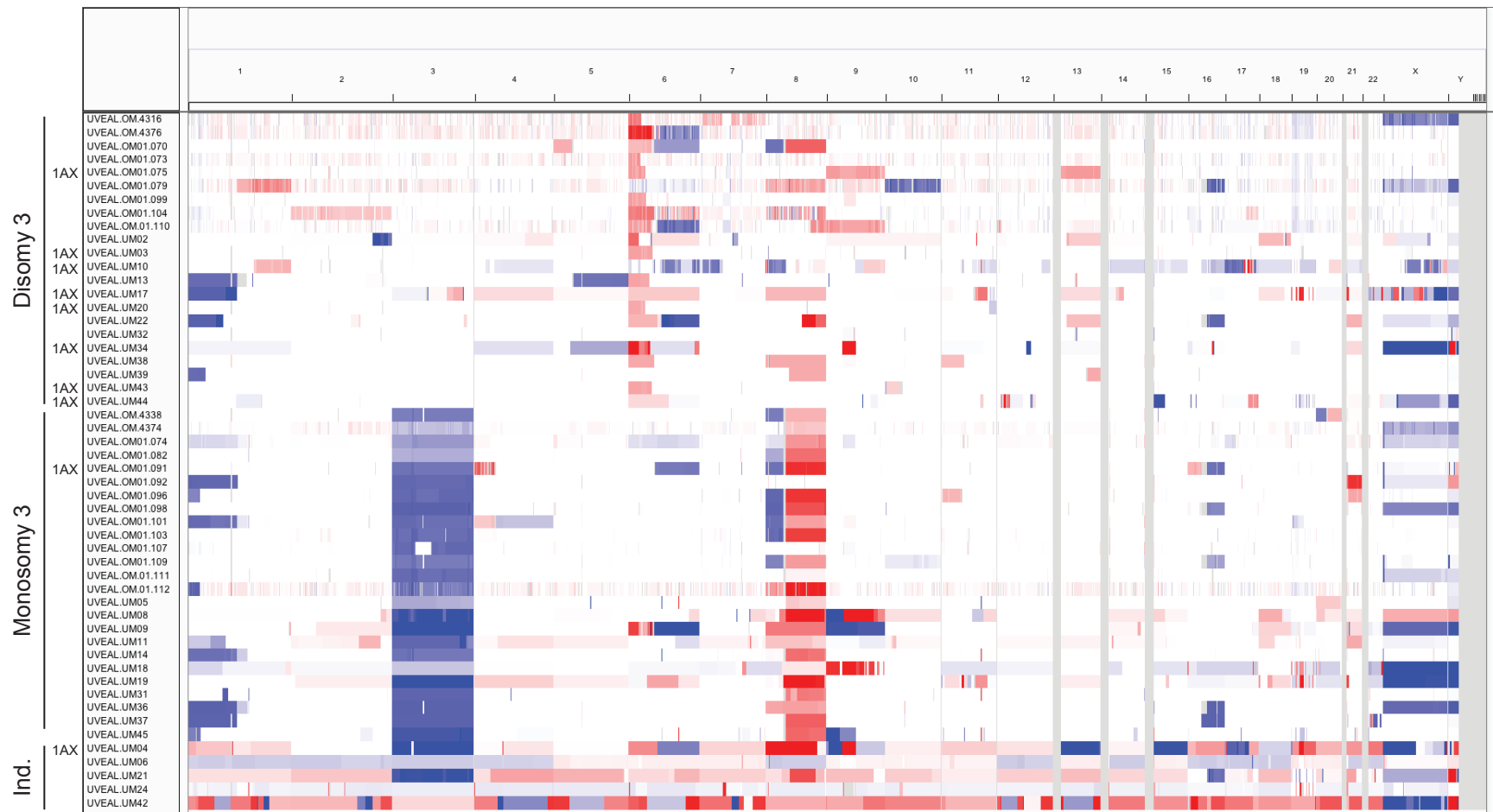

Supplement: S1 Fig — Coverage values from whole exome sequencing were converted into segmentation files and visualized using IGV. Samples are ordered by chromosome 3 status, or labeled Indeterminate (Ind.) due to noise across the genome. 1AX indicates samples with an EIF1AX mutation. (PDF) [file pone.0178189.s008.pdf]

Supplemental Fig. 3

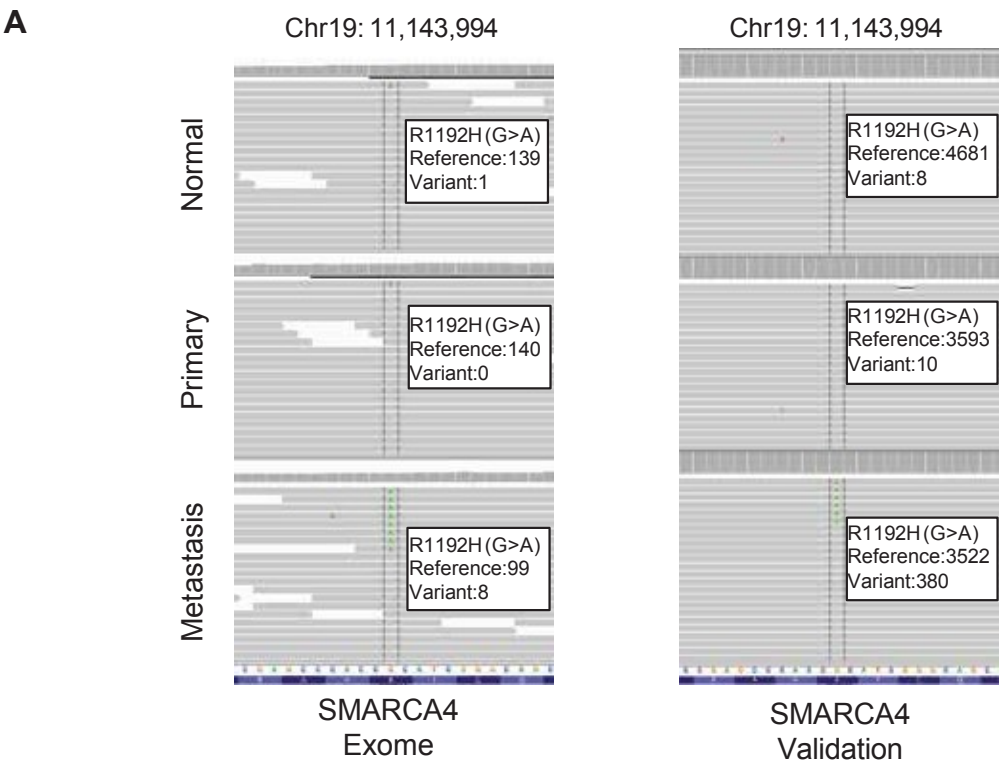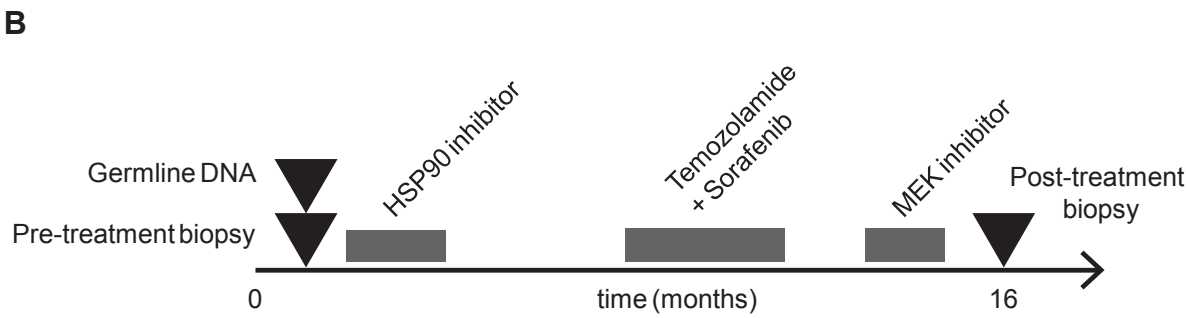

Supplement: S3 Fig — (A) IGV screenshot of SMARCA4 mutation from exome sequencing and targeted validation of UM45. (B) Trio 2 biopsy and treatment are indicated across time course. (PDF) [file pone.0178189.s010.pdf]

Supplemental Fig. 4

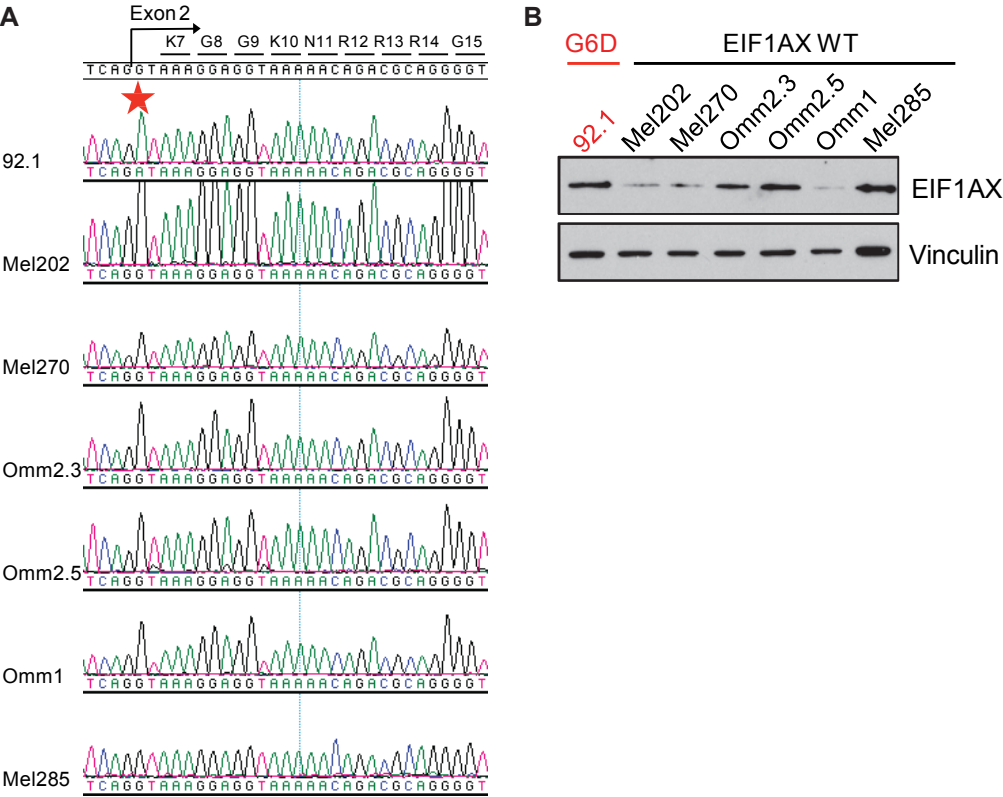

Supplement: S4 Fig — (A) Exon 2 sequencing trace displays putative EIF1AXG6D mutation in 92.1 cell line. (B) Immunoblot analysis of EIF1AX protein levels. (PDF) [file pone.0178189.s011.pdf]

Supplemental Fig. 5

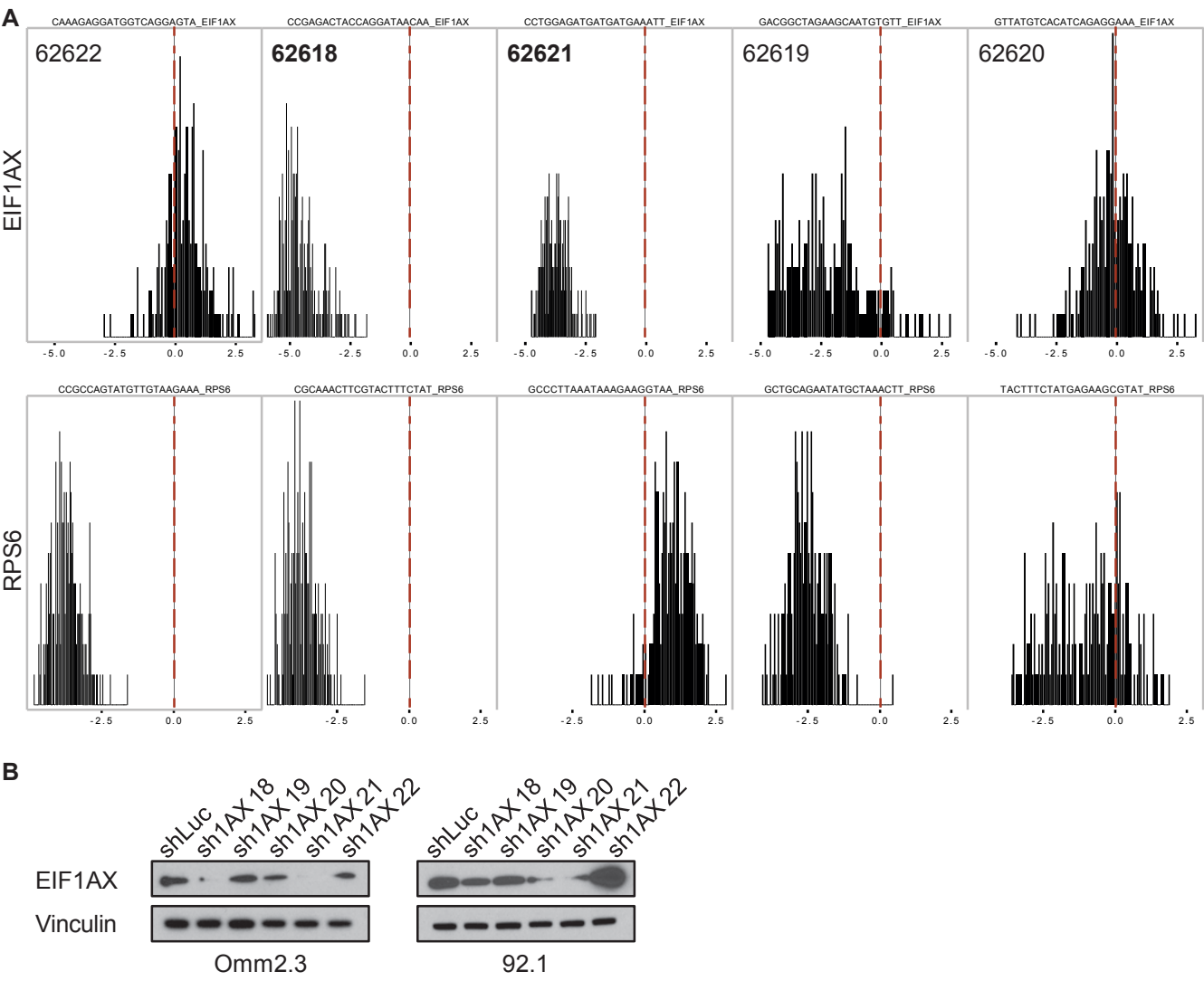

Supplement: S5 Fig — (A) Histograms represent shRNA level scores (normalized log fold change) for 5 EIF1AX (top) and 5 RPS6 (bottom) shRNAs from 216 cell lines in Achilles v2.4.3. Lower values represent more depletion indicating more dependency. EIF1AX shRNAs used in this study are bolded. (B) Immunoblot analysis of EIF1AX protein levels in 2 uveal melanoma cell lines expressing indicated shRNAs. (PDF) [file pone.0178189.s012.pdf]

Supplemental Fig. 6

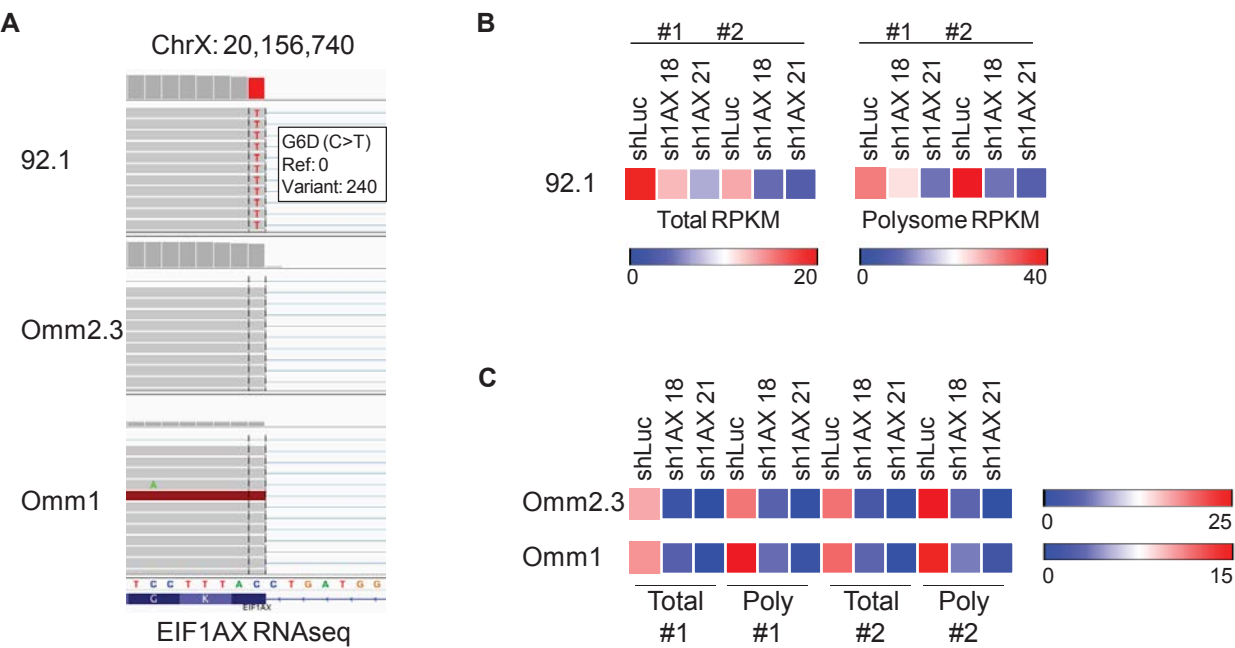

Supplement: S6 Fig — (A) IGV screenshot of EIF1AX exon 2 start indicates exclusive mRNA expression of the G6D variant in the 92.1 cell line. (B) Heatmap displays EIF1AX expression levels in RPKM for total and polysome-associated mRNA in 92.1 cells expressing indicated shRNAs. (C) As in (B), but for Omm2.3 and Omm1 EIF1AX-wild type cells. (PDF) [file pone.0178189.s013.pdf]
