# Supplementary material for: Systematic genomic and translational efficiency studies of uveal melanoma
Source: PLoS One. 2017 Jun 8;12(6):e0178189. doi: 10.1371/journal.pone.0178189 (PMC5464544; doi:10.1371/journal.pone.0178189)

Supplemental Fig. 2

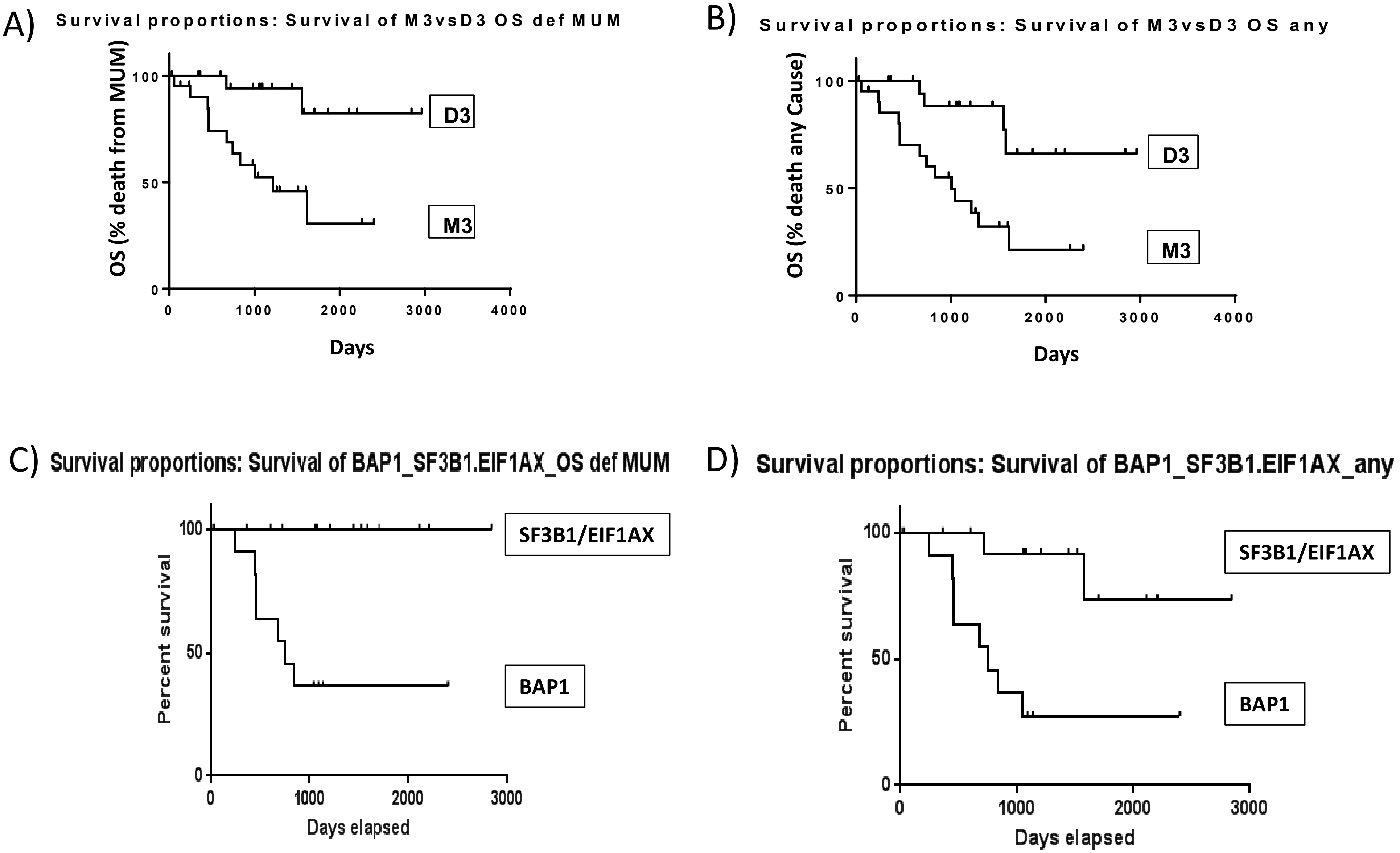

Supplement: S2 Fig — (A and B) Kaplan-Meier analysis showing primary uveal melanoma patients with evaluable OS data who had either monosomy 3 (n = 20) or disomy 3 (n = 23) tumors. OS reflective of death from metastatic uveal melanoma; the median survival was 1216 days in the monosomy 3 cohort and not reached in the disomy 3 cohort. Log-rank p value = 0.0034. HR = 6.9, 95% Cl (1.7 to 15.5) (B) As in (A), but overall survival reflective of death from any cause; the median survival was 1008 days in the monosomy 3 cohort and not reached in the disomy 3 cohort. Log-rank p value = 0.0034. HR = 4.5, 95% Cl (1.6 to 10.3). (C) Kaplan-Meier analysis showing primary uveal melanoma patients with evaluable OS data with tumors harboring a BAP1 (n = 14) vs. SF3B1 or EIF1AX (n = 14) mutation. OS reflective of death from metastatic uveal melanoma; the median survival was 744 days in the BAP1 mutant cohort and not reached in the SF3B1/EIF1AX mutant cohort. The Log-rank p value = 0.0008. HR = 13.7, 95% Cl (3.0 to 62.9). (D) As in (C), but OS reflective of death from any cause; the median survival was 744 days in the BAP1 mutant cohort and not reached in the SF3B1/EIF1AX mutant cohort. The Log-rank p value = 0.0022. HR = 7.5, 95% Cl (2.1 to 28.8). Note, sample UM 36 had both BAP1 and SF3B1 mutations, but was analyzed as a BAP1 mutant sample. (PDF) [file pone.0178189.s009.pdf]
